# Context-dependent selection as the keystone in the somatic evolution of cancer

**DOI:** 10.1038/s41598-020-61046-7

**Published:** 2020-03-06

**Authors:** B. Vibishan, Milind G. Watve

**Affiliations:** 1 Department of Biology, Indian Institute of Science Education and Research (IISER), Pune, India; 2 BILD Clinic, Deenanath Mangeshkar Hospital and Research Centre, Pune, India

**Keywords:** Cancer models, Evolutionary theory

## Abstract

Somatic evolution of cancer involves a series of mutations, and attendant changes, in one or more clones of cells. A “bad luck” type model assumes chance accumulation of mutations. The clonal expansion model assumes, on the other hand, that any mutation leading to partial loss of regulation of cell proliferation will give a selective advantage to the mutant. However, a number of experiments show that an intermediate pre-cancer mutant has only a conditional selective advantage. Given that tissue microenvironmental conditions differ across individuals, this selective advantage to a mutant could be widely distributed over the population. We evaluate three models, namely “bad luck”, context-independent, and context-dependent selection, in a comparative framework, on their ability to predict patterns in total incidence, age-specific incidence, stem cell number-incidence relationship and other known phenomena associated with cancers. Results show that among the factors considered in the model, context dependence is necessary and sufficient to explain observed epidemiological patterns, and that cancer evolution is largely selection-limited, rather than mutation-limited. A wide range of physiological, genetic and behavioural factors influence the tissue micro-environment, and could therefore be the source of this context dependence in somatic evolution of cancer. The identification and targeting of these micro-environmental factors that influence the dynamics of selection offer new possibilities for cancer prevention.

## Introduction

Mathematical models of somatic evolution in cancer have been in development for the past several decades, with a strong focus on mutational processes^1–6^. All cancers are necessarily a combination of different types of genomic changes including point mutations, aneuploidy and other chromosomal aberrations. The cancer phenotype has a large number of distinguishing characters, encapsulated by the notion of the “hallmarks” of cancer^7–9^. The wide range of characteristics that these hallmarks include makes it astonishing that so many alterations in cell properties come together in cancers purely out of chance.

Cancer is modeled as a multistage process but the dynamics of the multistage process is still largely unknown. The series of genetic or epigenetic changes have been considered to co-occur by chance alone by the ‘bad-luck’ paradigm. The clonal expansion paradigm assumes that every oncogenic mutation on the way to a cancerous phenotype causes the mutant clone to expand, and as the mutant population increases, the probability of a second oncogenic mutation increases proportionately^10^. Implicit in this theory is the assumption that every oncogenic mutation has a selective advantage over the normal cell. Since most changes involved in carcinogenesis relate to evading growth regulatory mechanisms, it is considered logical that any mutation that allows for such evasion will have a selective advantage. However, evidence has been accumulating over the past few years that the fitness advantage of a mutant is largely dependent on the tissue micro-environment^11,12^. Studies in mice^13^ and humans^14^ have demonstrated the effect of contingent factors, such as behavioural profiles and lifestyle parameters, on cancer progression. With changes in the lifestyle parameters, the age adjusted incidence for many types of cancers has increased significantly in recent decades (Supplemental Information from^15^). These findings provide clear indications that the selective forces which determine mutant clone fitness can vary considerably in response to many factors that vary across individuals, leading to *context-dependent clonal expansion* of potentially oncogenic mutants. The role of age induced changes in the microenvironmental selective forces in somatic evolution has been modeled as well as demonstrated^16–23^. But the role of individual differences in microenvironment at any given age has not been adequately incorporated by cancer models so far.

In this paper, we compare three modeling approaches, two of which explicitly include selection at different levels: (1) random mutagenesis, or the “bad luck” hypothesis, (2) context-independent selection (CIS) on mutant clones within individuals, and (3) context-dependent selection (CDS), where the selective forces vary depending upon the tissue micro-environmental context differentially in every individual. Genetic mutations are a fundamental aspect of multi-stage models of carcinogenesis. In our models however, we use the term mutation more broadly, to denote any change that is heritable within a given cell lineage, genetic or epigenetic.

Since well-curated data are available for human cancer incidence patterns through the SEER databases^24^, we develop models of these three processes, and compare their predictions with the epidemiological picture of cancer in the human population. A good working model of somatic evolution of cancer needs to explain the following epidemiological features:

1. Total and age-specific incidence: The pooled incidence of all cancer types lies well below 30%, while age-specific and cumulative incidence patterns show more variations between cancer types^24^. Interestingly, recent analyses have shown for several cancers that the age-specific incidence rates decline late in life in humans as well as in rodents^25–27^, in contrast with a previous belief of a monotonic power law increase in incidence with age^1,28^. Some theoretical explanations for this decline have been suggested^29,30^, but biologically interpretable and translationally useful insights are yet to be obtained. The late-life decline causes the cumulative incidence to saturate with age at a small percentage of the population size. No matter the lifespan, the proportion of cancer in the population can never reach 100%, but saturate at a finite limit.
2. Incidence vs cell number: The relationship between cancer risk and stem cell number (as the lifetime number of stem cell divisions) has been kept in the spotlight by recent work by Tomasetti *et al*.^5,6^ and its criticism by others^15,30^. Since good data are available on this relationship, if the three models make differential predictions about the relationship, it can be used to evaluate the models comparatively. Empirical data on this relationship of mutation rate versus incidence are less common, as mutation rates are difficult to measure reliably. Therefore it is difficult to use this relationship as a testable prediction although some insights are possible^29,31^.
3. Non-mutagenic carcinogens: There are several agents, including hormones and growth factors that increase cancer risk without affecting the basal mutation rate^32^. The activity of these agents, and their signature in epidemiological patterns, are both important in building a complete framework of explaining cancer etiology.
4. Peto’s paradox and similar observations: This relates to the incidence-cell number relationship, as cancer risk is seen not to scale with body size or cell number across species^33^, but may correlate with the latter within a species^34^. A wide range of explanations have been offered for this observation^35^, and in a modeling context, these explanations can come from the model itself (intrinsic), or involve extrinsic factors, such as evolved cancer defenses, that are not part of the basic model framework.

### The “bad luck” model

This hypothesis assumes that the required set of driver mutations accumulate in a cell by chance alone. This may happen over a period of time, or in a single large-scale event, like chromothripsis^36^. Regardless of whether mutations accumulate sequentially or otherwise, the “bad luck” model does not assume selection of any kind on mutations over the course of somatic evolution. Typical bad luck model is given by Calabrese and Shibata^37^ and Zhang *et al*.^30^. We use the model for life time probability of cancer parameterized by age,

1 $${P}_{can}=1-{(1-{(1-{(1-p)}^{logS+d.a})}^{k})}^{S}$$

where *S* is the numbers of stem cell, *d* the stem cell division rate, *k* the number of driver events required for cancer onset and *p* as the mutation rate per division. We use *S* for a steady state stem cell number and *n* (see below) for total number of stem cell divisions up to a given age.

**Figure 1:**
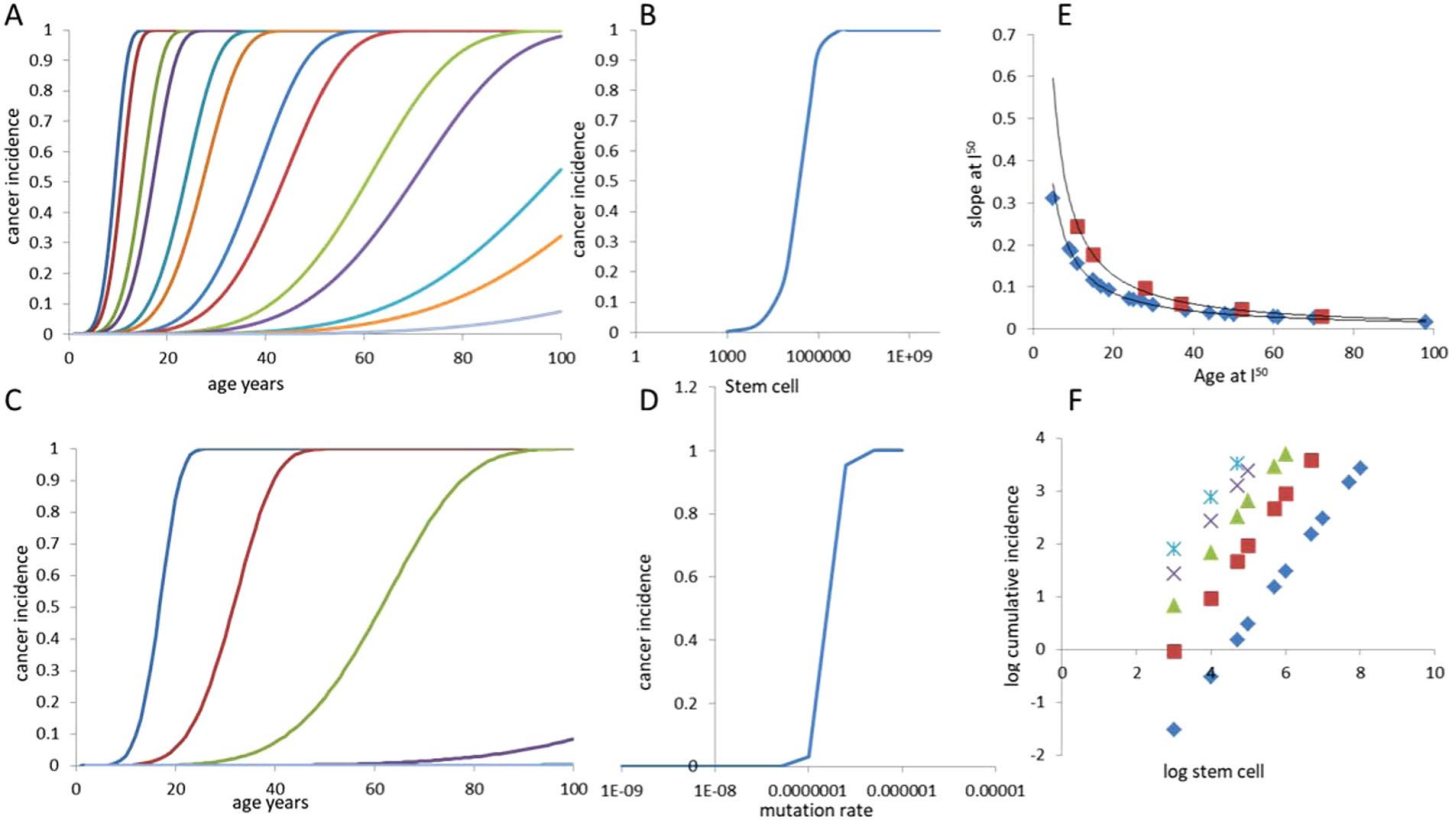
The nature of relationships between age, stem cell number, mutation rate and cancer incidence by the bad luck model. The cumulative incidence of cancer increases with age in a threshold relationship. (**A**) The stem cell number affects the threshold age, but in all cases the trend saturates only at 100% incidence. (**B**) At any given lifespan, the stem cell number holds a threshold relationship with cancer incidence. For tissues with stem cell numbers below the threshold, the cancer probability is close to zero and above the threshold it rises to almost unity. (**C**) The mutation rate also influences the threshold age for a given cancer type and given a fixed lifespan, the mutation rates holds a threshold relationship with incidence (**D**). The age threshold and the sharpness of the threshold measured by the slope at 50% cumulative incidence (I_50_) have an inverse relationship (**E**) shown at k = 5 and 8 respectively. If we take only those cancers for which the threshold lies beyond the maximum lifespan, the relationship of stem cell number with lifetime cancer incidence on a log-log plot is linear (**F**) with the slope = 1. Lifespan and other parameters change the position of the line, but the slope remains unaltered. Shown in the graph are analytical results. Simulations at population size 10,000 and above follow the analytical predictions very closely. Parameters: For A and B, p = 10^−6^, n ranges from (curves right to left) 10^3^ to 10^10^. For C and D, n = 10^9^ and p ranges from (curves left to right) 10^−6^ to 10^−9^. In all results displayed here k = 5 and d = 1000.

From Eq. 1, the cancer incidence shows a threshold relationship with the number of stem cells as well as the mutation rate. The cumulative incidence also increases with age in a threshold relationship saturating only at 100% (Fig. 1). The sharpness of the threshold as measured by the slope of the curve at the point of inflection (i.e. at cumulative incidence of 50%) is inversely proportional to the threshold age. The age-slope inverse relationship is not affected by stem cell number and mutation rates but at larger *k* the threshold becomes sharper. The threshold relationship has been obtained by earlier models as well^2^ but its implication in terms of testable predictions of the models have not been discussed or used. Because of the threshold relationship, if cancers were not lethal, then we would have obtained a bimodal relationship of incidence with stem cell numbers. For tissues with stem cell numbers above the threshold, the incidence would have been near 100% and for tissues with low numbers close to none. Since cancers are fatal, we would expect that the first tissue to exceed the threshold would kill almost 100% of the population before cancers could develop in any other tissues. Since we do not see such patterns in the population, we are forced to restrict ourselves to the part of the parameter space in which the threshold for any tissues lies beyond the maximum lifespan. Under these conditions, the incidence increases monotonically with age in a power law relationship with no signs of late age decline. The expected relationship between stem cell number and cancer incidence across different tissues is a straight line on a log-log plot with a slope very close to unity. Lifespan and mutation rates affect the position of the line but the slope remains unaltered. This can be taken as a testable prediction of the bad luck model.

### Models with selection on mutants

For models that involve selection, we choose to explore the effects of CIS and CDS through a simulation-based stochastic framework.

We use a linear process to model the sequential accumulation of mutations in a population of stem cells. We begin by considering the development of a generalized tissue compartment in each organism starting from one stem cell, with mutation rate per cell generation per locus, *p*, growing logistically to a carrying capacity. Conceptually the carrying capacity is the same as standing stem cell number in adult tissue *S*. We use the discrete logistic equation

2 $${m}_{i.t}={m}_{i.t-1}+{m}_{i.t-1}\,\ast \,{g}_{i}\,\ast \,(\frac{S-{\sum }_{i=0}^{k}{m}_{i,t-1}}{S})-{m}_{i,t-1}\,\ast \,d$$

Here, *m*_*i,t*_ is the size of the *i*^th^ mutant population at time, *t*, with *i* = 0 being the non- mutant cell population, *g*_*i*_ the corresponding logistic growth rate*, d* the common death rate, and *k* is the threshold number of oncogenic mutations required for cancer onset. As the organism develops into an adult, net growth in the stem cell compartment saturates, but reaches a dynamic equilibrium between cell death and renewal. The stem cell population can be reduced, either by death of stem cells or differentiation, as reflected by *d* in Eq. 2. The replacement of the lost cells by either mutants or non-mutants is a function of their growth rates. We simulate new mutation events stochastically; the probability of at least one cell mutating is given by $1-{(1-p)}^{{m}_{i,t}}$, and if this probability exceeds a random number between 0 and 1, a new (*i* + 1)^th^ mutant population is initiated. Each new oncogenic mutation could give a growth advantage over older cell populations, leading to successive cycles of clonal expansion in which the newer population gradually replaces older cells through competitive exclusion. We simulate this linear evolution process until *k* mutations have been accumulated, which is the assumed threshold for cancer onset. Death of the individual occurs either at cancer onset when the *k*^th^ mutation occurs, or at the end of the assumed lifespan of 100 years, whichever happens first. This simulation is repeated independently for a population of 10000 individuals, and the population-level cancer incidence is recorded, along with the age of onset.

### Choice of parameter range

In order to standardize the discrete logistic simulation, we assume the time unit to be one day per logistic growth step. Most human organs complete development and maturation with in the first 10–20 years of the lifespan, and the final carrying capacity achieved is the adult stem cell number, ranging between 10^6^ and 10^11^ across different tissues (Supplementary Material from^5^). Given the final population size and the time taken to reach it, a simple calculation based on the logistic equation shows the required growth rate for a non-mutant stem cell to be in the range of 0.00383 to 0.0131. Starting from the non-mutant growth rate, *g*_0_, growth rates are assumed to increase linearly for each subsequent mutant population, with the slope given by $\varDelta g=\frac{{g}_{k}-{g}_{0}}{k}$. Ranges of stem cell number and mutation rate are retained as in the “bad luck” model.

### The context-independent selection (CIS) case

While the clonal expansion theory introduced the notion of selective advantages to oncogenic mutants, it makes the implicit assumption that identical mutations have the same selective advantage in every individual in which they occur; stated otherwise, individuals do not differ in their propensity for mutant clonal expansion. To capture this in the CIS case, we use the same slope, *Δg* for all individuals in the simulation.

### The context-dependent selection (CDS) case

As argued earlier, it is becoming increasingly clear that the competitive outcomes of identical mutations can depend strongly on the micro-environmental context in which cell competition occurs. In order for selection on mutants to be context-dependent in our model, we randomize the slope, *Δg* over the population from a given normal distribution. Each individual begins with the same *g*_0_, but the progression of growth rates is randomized across individuals, such that individuals with large *g*_*i*_ would progress faster towards cancer onset, while those with small, or negative values of *g*_*i*_ would never progress to a cancerous state as the mutant gets selected against. This produces variation across individuals for cancer propensity.

### Predictions from the selection models

**Figure 2:**
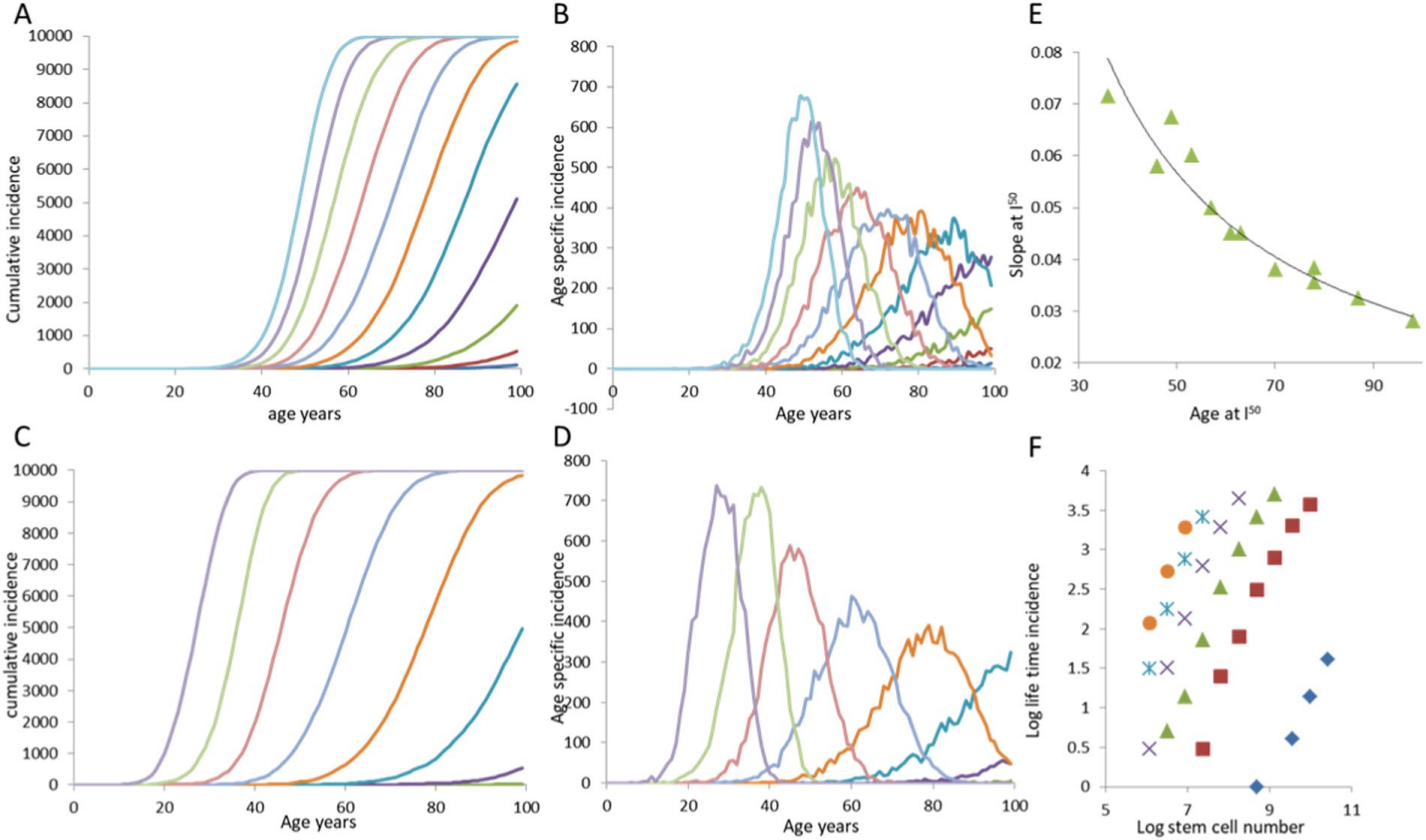
The nature of relationships between age, stem cell number, mutation rate and cancer incidence by the CIS model. (**A**) Similar to the bad luck model, there is a threshold age at which the incidence transits from near zero to near 100%. Saturation of cumulative incidence happens only when the incidence approaches 100%. The threshold age is affected by the stem cell number (**A**,**B**) as well as the mutation rate (**C**,**D**). The threshold age and the slope at I_50_ holds an inverse relationship (**E**) similar to the bad luck model. (**F**) For the parameter range in which the threshold lies much beyond the lifespan, the log-log relationship between stem cell number and cumulative incidence is linear with a slope > = 1. For A and B, p = 5.603 10^−9^, n ranges from (curves right to left respectively) 10^6^ to 10^10.5^. For C and D, n = 1.785 10^8^. p ranges from (curves left to right) 10^−6.5^ to 10^−10.5^. Growth rates progress linearly in the general form, ${g}_{i}$ = 0.007 (i + 1), where i = 0 to k and k = 5. Here, *Δg* = 0.007. k = 5 for all.

**Figure 3:**
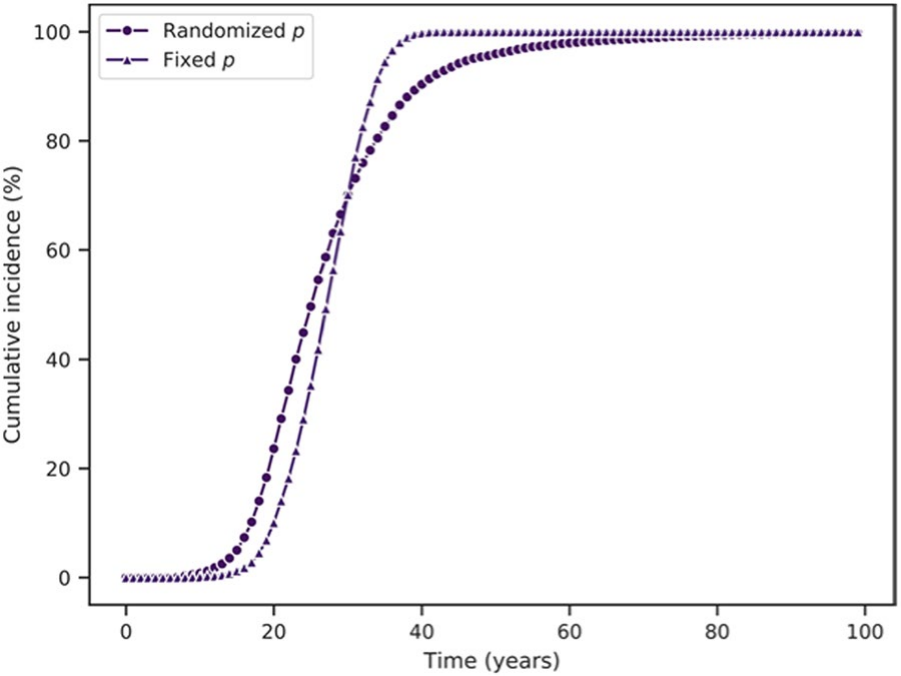
Age trend in cumulative incidence with randomized vs fixed p for the population in the CIS case; giving a distribution to p does not reduce the saturation of incidence from 100%, but slows the transition to 100%. In the randomized case, p values were drawn from a uniform distribution with range [3.775 ∗ 10^−11^, 3.06 ∗ 10^−7^], while for the fixed case, p = 3.06 * 10^−7^; for both, n = 1.785 * 10^8^ and k = 5.

Under the assumption of CIS, the incidence of cancer shows a strong threshold relationship with age, saturating only at 100% (Fig. 2). The age-specific incidence declines only when the cumulative incidence approaches 100%. As with the “bad luck” model, incidence has a threshold relationship with both *n* and *p*. Where the incidence of cancer is near the realistic range, for small values of *p* and *n*, the late-life decrease in incidence is not observed. In this range of parameters the log-log plot of stem cell numbers with cancer incidence gives a straight line with slope > = 1. Giving a distribution to *p* in the realistic range reduces the sharpness of the incidence threshold with age, but saturation of incidence remains at 100% (Fig. 3). By most of these predictions it is difficult to resolve the CIS model from the bad luck model except that the log-log plot of stem cell number and life time incidence may have a slope >1 in CIS model.

**Figure 4:**
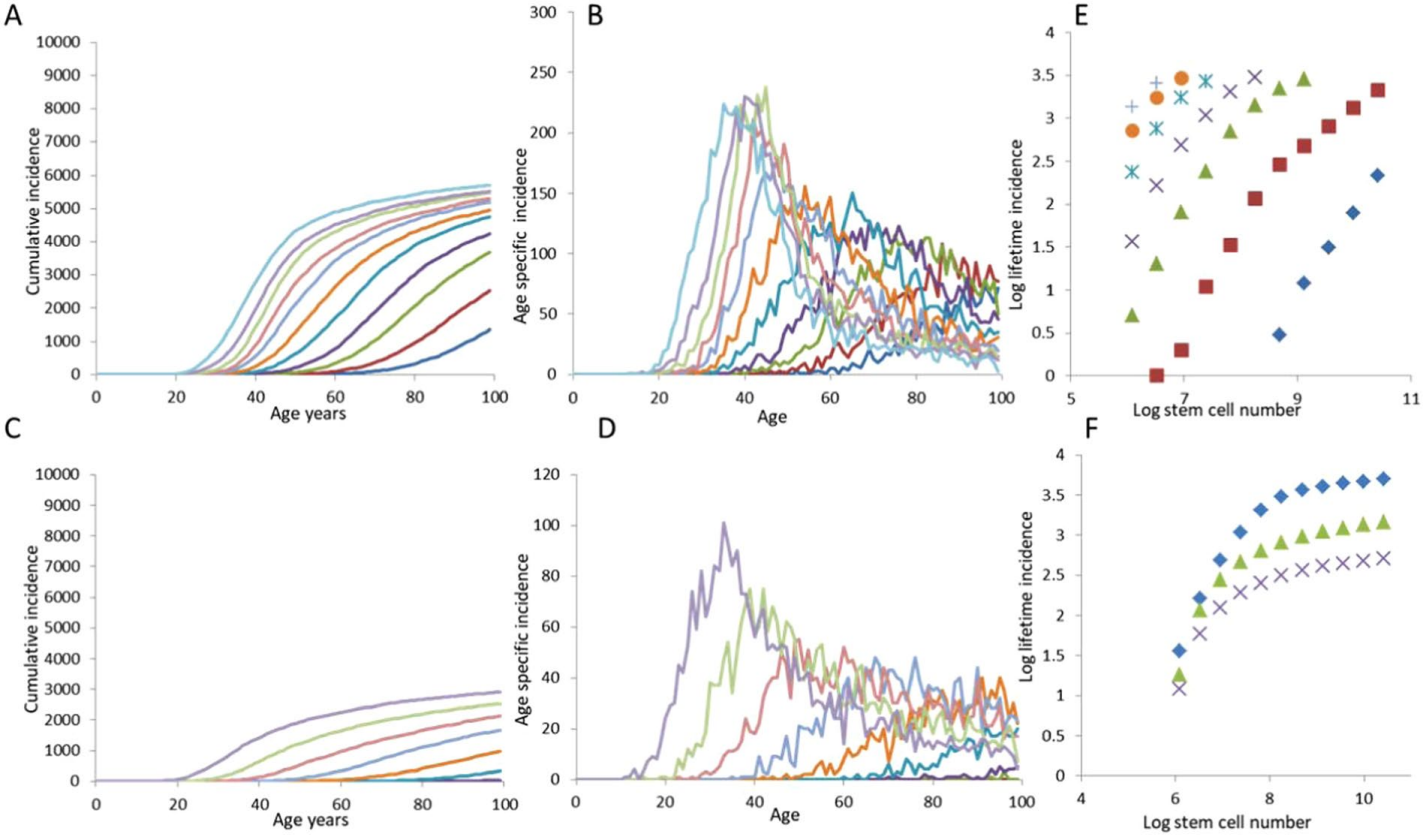
The nature of relationships between age, stem cell number, mutation rate and cancer incidence predicted by the CDS model. In this model the cumulative incidence can saturate much below 100% depending upon the distribution of *Δg*. The age incidence curve at different n (**A**,**B**) and different mutation rates (**C**,**D**) show an early saturating trend rather than a threshold phenomenon. The log-log plot between stem cell number and life time incidence may be saturating or linear with a slope < = 1 (**E**). With a reduction in the mean *Δg*, the cell number incidence curve saturates early (**F**). All parameters similar to Fig. 2.

As opposed to the CIS model, the CDS model produces a saturating trend in cumulative incidence that saturates at a level much lower than 100% (Fig. 4). The early saturation happens when a proportion of the population has relative growth rates zero or negative. This assumption is theoretically as well as empirically sound as a mutant can have positive or negative fitness^19^. This is an important feature of the CDS model, as it allows the model to generate more realistic patterns in age-specific cancer incidence. The saturation occurs because propensity for clonal expansion varies across individuals; cancer progression occurs very quickly in some individuals, and not at all in others. Of the three models analyzed so far, only the CDS model captures a trend similar to the late-life decline observed in many cancers in humans (Fig. 4). The stem cell number and incidence relationship on a log-log plot, in this model, can have a slope <1 and often the relationship shows a saturating curve rather than a straight line. These predictions of the CDS model contrast the bad luck and CIS model and can be used as differential testable predictions. The major differential predictions of the CDS model arise from the *Δg* distribution which implies that somatic evolution of cancer in this model is mainly selection limited rather than being mutation limited.

### Sensitivity of predictions

We relax the quantitative and qualitative assumptions of the model to test whether or not the results are artifactual, contributed by any specific assumption.

We assumed *k* to be constant for a given cancer type, however across cancer types the estimated *k* varies^38,39^. An increase in *k* does not qualitatively affect the threshold phenomenon in the bad luck and CIS models but shifts the threshold age to the right. For a given threshold age the threshold is sharper for a higher *k*. Randomizing *k* between the range 2 to 10 (supplementary figures S2.1 to S2.4) increases the scatter but does not introduce any pattern by itself.

To test whether the assumption of a normal distribution for *Δg* is critical for the results, we reevaluated the predictions of the CDS model for a Gumbel and uniformly-distributed *Δg* progression. We find that the shape of the distribution does not affect the saturation of cumulative incidence, or the late-life decline in age-specific incidence predicted by the model, although the shape of the age curve changes (Supplementary Material, S1 Figures). We assume generally that *Δg* is constant for a given individual, but relaxing this assumption by giving a distribution to *Δg* within each individual would not change the results qualitatively. In such a case, the lowest *Δg* within an individual would be the rate-limiting step.

Both our selection models assume a linear somatic evolution process, by which the tissue has only one evolving lineage of cells at a time. Although we do not explicitly incorporate branched and polyclonal evolution^40,41^, our framework is, in principle, compatible with them and we do not expect a qualitative change in the predictions of CIS and CDS models. In fact, a branched evolution process allows for a wider range of ecological interactions between sub-clonal populations, mutualistic or otherwise^42,43^. Such interactions again lend into a selection-driven framework rather than a “bad luck” driven one.

Somatic cell populations are potentially susceptible to mutational meltdown, and a large number of somatic mutations are in fact known to be deleterious. Earlier analysis of the effect of passenger mutations has shown that accounting for them in somatic evolution delays overall incidence of cancer in the population^2^. However, from our CIS case, we see that when total incidence in the population decreases, most cases of cancer are concentrated later in life, similar to the delay in incidence noted before^2^. This pattern is not compatible with the observed late-life decline in incidence. We expect therefore that mutational meltdown due to deleterious passenger mutations is insufficient to explain saturation due to late-life decline in incidence.

### Differential testable predictions of the models

Summarily the testable predictions of the three classes of models with reference to the epidemiological features that we defined in the beginning are as follows,

1. The bad luck model and the CIS model predict a threshold relationship with age. Saturation in the incidence is not predicted below 100% incidence. In CDS model, saturation at a level well below 100% is possible depending mainly on the distribution of *Δg*. Nevertheless, a power law like relationship is also possible when the incidence is much below the saturation level. Different types of cancers show different shapes of age incidence curves. The power law like relationships can be explained by any of the models at certain parameters, but the saturating relationship with late life decline even at a small cumulative incidence is not described by the bad luck or the CIS model unless some extrinsic causes or statistical distributions are incorporated^30^.
2. The relationship between stem cell number and cancer incidence is differentially predicted by the three models. While the bad luck models expects a linear relationship with a slope = 1 on a log-log plot, the CIS expects a slope > = 1. The CDS, in contrasts expects a saturating relationship or one with a decreasing slope. In the part of the curve that may be approximated to a linear relationship, the slope is < = 1. Although Tomasetti and Vogelstein^5,6^ claim the cell number incidence relationship to be linear on a log-log scale, the slope is substantially smaller than one. Further our analysis (supplemental material S3) of the same data reveals that even on a log-log scale, a saturating curve describes the relationship better than a straight line. This supports the CDS model and contradicts the predictions of bad luck and CIS models.
3. The bad luck and the CIS models offer no direct explanation for non-mutagenic carcinogens, whereas the CDS model suggests that they can work by affecting the *Δg* distribution by altering microenvironmental conditions. Even carcinogenic exposures such as ionizing radiations that were thought to affect mutagenesis alone, are now shown to act also by affecting the selective environment^44^.
4. The Peto’s paradox is based on the assumption that the somatic evolution of cancer is mutation limited. The bad luck and CIS models are primarily mutation limited. Therefore they need an additional explanation for the Peto’s paradox such as evolved anti-cancer defenses in organisms with large body sizes and longer lifespans. In CDS, on the other hand, mutations are not limiting and therefore a relationship with the body size or cell number is not expected through most of the size range. Our model is compatible with the argument that Peto’s paradox can be explained by invoking selection limited cancer evolution^33–35,45^.

The model prediction matrix suggests that the bad luck model and the CIS model face many incompatibilities with real life data leading to rejection of these models, whereas the distribution of *Δg* in the population incorporated in the CDS model is sufficient to predict the observed patterns in the relationships between age, stem cell number and cancer incidence. Among the factors considered in the model, context dependent selection is necessary to simultaneously explain different types of shapes of the age-incidence curves, saturation at a lower incidence level, a saturation relationship between stem cell number and incidence, non-mutagenic carcinogens and Peto’s paradox. While these phenomena have been separately explained by different models with different set of assumptions, simultaneous compatibility to all these phenomena is unique to context dependent selection among the models suggested so far.

## Discussion

On the whole, the better prediction profile of the CDS model stems from the distribution of *Δg* in the population. The gradual late-life decline in incidence is because of the left-hand tail of the *Δg* distribution, where in some individuals mutants have very small, zero or negative fitness.

The role of age induced changes in the selective microenvironment is recognized by some of the prior cancer models^16–19^ and supported by empirical evidence^20–23^. However, these models do not address the late age saturation and decline. Incorporation of individual differences at any age class is essential to explain these patterns. Our model mainly considers individual differences in the selective environment but is compatible with the age induced changes as well. Some types of leukemia exhibit an early life peak in incidence and a second gradual late life rise^19,46^. A combination of individual differences and age related changes in microenvironmental conditions will be able to explain such a phenomena better. The age dependent models differentiate between physiological aging and chronological aging^18^. Within any population, individuals differ in physiological decline at a given chronological age, which necessitates incorporation of *Δg* distribution at a given chronological age in these models too. It is possible that the factors influencing the evolution of aging influence the mechanisms that determine the mean, distribution and temporal trends in *Δg* distribution. In addition, smaller animals have shorter cell generation times and faster turnover rates and as a result different species have different age-incidence curves.

The individual variation in selection could be attributed to the micro-environment in the tumor or pre-cancerous niche, and includes all the factors that determine the selective advantage of oncogenic or pre-cancerous mutants. Cellular interactions with the extra-cellular matrix (ECM) are known to be important in tissue homeostasis^47^ and the growth of cancer cells^12^. The tissue micro-environment is also a key player in the association between obesity and cancer risk, where obese ECM differs from normal ECM both biophysically^48^ and functionally^49^. The immune system also acts as an important mediator in cancer-related processes; a wide range of immune cells including fibroblasts and lymphocytes are known to influence cancer progression under various conditions^11^. Cancer is increasingly seen as an overactive or abnormal wound healing process^8^ and signals of wound healing pathways might affect the growth rates of driver mutants. *In vitro*, the IGF-II concentration in culture media was found to markedly alter the selective advantage of an IFG-II over-expressing mutant in cell competition^50^. Pre-cancerous cell lines across cancer stages also show different growth properties depending on growth factor concentration in the culture medium^51^. Similarly, levels of estrogen and progesterone are known to play important roles in regulating mammary cell proliferation^52^, secretion of ECM factors, tumor-stroma interactions^53^ and growth factor production^54^. In intestinal tissue, colitis gave specific selective advantage to P_53_ mutants^55^. More recently, experiments have been reported in which behaviourally-enriched environments or physical exercise seemed to show cancer-suppressive effects^13,14^ that correlate with levels of particular growth factors. Studies in mice have also shown substantial variation in tumor sizes induced by identical genetic clones across individuals^56^. These observations together suggest that CDS factors from the microenvironment, be they genetic-epigenetic, biophysical, ECM-derived, GF-derived, hormonal or immunological, could play a limiting role in cancer progression. Identifications of the factors governing selective advantage of driver mutations needs to be a focus of future cancer research which can potentially lead to novel lines of cancer prevention strategies.

## Supplementary information

Supplementary information.
